# Hepatitis B Virus X Protein-Induced Serine Protease Inhibitor Kazal Type 1 Is Associated with the Progression of HBV-Related Diseases

**DOI:** 10.1155/2019/9321494

**Published:** 2019-05-22

**Authors:** Chengliang Zhu, Huan Han, Jie Li, Limin Xu, Fang Liu, Kailang Wu, Xinghui Liu

**Affiliations:** ^1^Department of Clinical Laboratory, Renmin Hospital of Wuhan University, Wuhan, Hubei 430060, China; ^2^Department of Clinical Laboratory, Shanghai Gongli Hospital, The Second Military Medical University, Pudong New Area, Shanghai, China; ^3^The State Key Laboratory of Virology, College of Life Sciences, Wuhan University, Wuhan, Hubei 430072, China

## Abstract

**Objective:**

Hepatitis B virus (HBV) causes inflammation of the liver and is the leading cause of both liver cirrhosis (LC) and hepatocellular carcinoma (HCC). Serine protease inhibitor Kazal type 1 (SPINK1) is an acute-phase response protein that is overexpressed in liver cancer tissue. This study investigated the clinical value of SPINK1 with regard to the diagnosis of HBV-related diseases and its regulatory mechanism.

**Methods:**

Serum levels of SPINK1 in HBV-infected patients and healthy participants were detected by enzyme-linked immunosorbent assay (ELISA). Reverse transcription quantitative real-time polymerase chain reaction (RT-qPCR) and western blotting were used to detect differential expression of SPINK1 mRNA and protein in HepG2 and HepG2.2.15 cells. The HBV infectious clone pHBV1.3 and its individual genes were cotransfected into HepG2 cells with the SPINK1 promoter coupled to a luciferase reporter; luciferase activity was measured, and the expression levels of SPINK1 were examined.

**Results:**

Serum SPINK1 levels of HBV-infected patients were significantly higher than those of healthy participants, and the serum levels of SPINK1 in patients who tested positive for HBeAg were significantly higher than those in patients who tested negative for HBeAg. The serum SPINK1 levels of patients with LC or HCC were markedly higher than those of patients with chronic hepatitis. The HBV X protein (HBx) activated the SPINK1 promoter to upregulate expression of SPINK1 at both mRNA and protein levels.

**Conclusions:**

HBV enhances expression of SPINK1 through X. SPINK1 levels are increased during progression of HBV-related diseases and might be utilized as a biomarker for the diagnosis of HBV-related diseases.

## 1. Introduction

Hepatitis B virus (HBV) infection is a serious public health problem worldwide. Approximately 250 million people have chronic HBV infection; of these cases, approximately 20% to 30% develop into liver cirrhosis (LC). Moreover, approximately 2% to 5% of cirrhosis cases progress to liver cancer [1, 2]. The gene serine protease inhibitor Kazal type 1 (SPINK1), also known as tumor-associated trypsin inhibitor (TATI) or pancreatic secretory trypsin inhibitor (PSTI), is located on chromosome 5q32, approximately 7.5 kb long, and contains four exons [3]. SPINK1, a secretory peptide composed of 56 amino acids, belongs to the Kazal-type serine protease inhibitor family, the main roles of which are to inhibit the activity of numerous pancreatic serine proteases. In addition, SPINK1 appears to participate in tumorigenesis [4–7]. HBV and hepatitis C virus (HCV) upregulate expression of serine protease inhibitor Kazal (SPIK), and SPINK1 is overexpressed in HCV-positive hepatocellular carcinoma (HCC) and thus is a promising prognostic marker for this cancer [8–10].

In a preliminary study, we screened for differentially expressed genes between control HepG2 cells and HepG2.2.15 cells integrated with the entire HBV genome and found that expression of SPINK1 was increased by more than 40-fold in HBV-infected HepG2.2.15 cells (data not shown). Although it is known that HBV is a major cause of HCC [2, 11], the mechanism by which HBV regulates SPINK1 expression remains unclear. This study investigated the effect of HBV infection on SPINK1 expression, analyzed the relationship between serum SPINK1 levels and the progression of HBV-related diseases, and explored the molecular mechanism underlying the regulation of SPINK1 expression by HBx.

## 2. Materials and Methods

### 2.1. Participants

A total of 248 patients with a clinical diagnosis of HBV infection from Renmin Hospital of Wuhan University (Wuhan, China) from January 2015 to November 2018 were included in the present study. The patients were divided into three groups according to the results of clinical biochemistry tests, computed tomography (CT), magnetic resonance imaging (MRI), and pathology examinations as follows: 104 patients with chronic hepatitis B (CHB) defined by persistent HBsAg positivity for > 6 months [12] (78 men and 26 women with a mean age of 40.5 ± 12.7 years), 82 patients with LC (60 men and 22 women with a mean age of 47.7 ± 15.3 years), and 62 patients with HCC (48 men and 14 women with a mean age of 55.8 ± 17.5 years). None of the patients had other viral infections, including HCV, HDV, or HIV. A total of 124 healthy participants (90 men and 34 women with a mean age of 42.2 ± 14.6 years) were included as a control group. The Ethics Committee of Renmin Hospital of Wuhan University approved this study, and all participants signed an informed consent document.

### 2.2. Cell Culture and Transfection

HepG2 and HepG2.2.15 cells were cultured in Dulbecco's Modified Eagle's Medium (DMEM; Gibco) containing 10% FBS and 100 U/ml penicillin-streptomycin in an incubator at 37°C and 5% CO_2_. HepG2 cells were seeded in 6-well or 24-well plates and were transfected with Lipofectamine 2000 transfection reagent (Invitrogen) according to the user's manual when reaching 80% confluence. Briefly, HBV infectious clone pHBV1.3, eukaryotic expression plasmids containing all of the HBV genes (pCMV-S, pCMV-E, pCMV-C, pCMV-X, and pCMV-P), and Lipofectamine 2000 were diluted in DMEM medium at room temperature for 20 min [13]. The prepared transfection solution was then added to the HepG2 cell culture medium, and the cells were cultured in a CO_2_ incubator.

### 2.3. Reverse Transcription Quantitative Real-Time PCR (RT-qPCR)

HepG2 and HepG2.2.15 cells were collected, 1 ml of Trizol^R^ reagent (Invitrogen) was added, and total RNA was extracted according to the user's manual. cDNA was synthesized from 1 *μ*g of total RNA using reverse transcription with M-MLV (Promega) in a volume of 20*μ*L; the cDNA was then diluted 10 times with DEPC water, and real-time fluorescence quantitative PCR amplification was performed using a Roche LightCycler 480 (Roche) in a reaction mixture of 20 *μ*L containing 10*μ*L SYBR Green qPCR Master Mix (DBI Bioscience), 0.5 *μ*M of each PCR primer, 3 *μ*L of diluted template, and RNase-free water to complete the 20 *μ*L volume. GAPDH was used as the internal reference. The upstream and downstream primers for SPINK1 were 5′-AACACTGGAGCTGACTCCCT-3′ and 5′-ATCAGTCCCACAGACAGGGT-3′, respectively. The PCR reaction was performed at 95°C for 10 minutes, followed by 35 cycles of 95°C for 15 seconds and 58°C for 30 seconds. The relative expression level of SPINK1 mRNA was expressed as 2^-∆∆Ct^. The experiment was repeated three times.

### 2.4. Western Blotting

Total protein was extracted from cells using RIPA lysis buffer (50 mM Tris-HCl, 150 mM NaCl, 1% Triton X-100, 1 mM EDTA, 10% glycerol, and protease inhibitor cocktail (Roche), pH 7.4) and quantified using BCA Protein Quantitation Assay Kit (Pierce). Samples of 50 *μ*g were separated by sodium dodecyl sulfate polyacrylamide gel electrophoresis (SDS-PAGE), and the protein bands were transferred to a nitrocellulose (NC) membrane. After blocking with phosphate-buffered saline (PBS) containing 0.1% Tween 20 (PBST) for 2 hours, the primary anti-SPINK1 antibody (Abcam, ab188307, diluted 1:2000) or anti-beta actin antibody (Sigma, diluted 1:5000) was added and incubated at 4°C overnight. The membrane was washed three times with PBST buffer, followed by the addition of a horseradish peroxidase-conjugated secondary antibody (Sigma, diluted 1:5000), incubation at room temperature for 1 hour, and three washes with PBST buffer, avoiding light. The enhanced chemiluminescence (ECL) method was used for development, followed by fixation and photography.

### 2.5. Luciferase Assay

Transfected HepG2 cells were cultured for 48 hours and then lysed using lysis buffer (Promega). A 20 *μ*L aliquot of the cell lysate and 100 *μ*L of luciferase substrate (Promega) were mixed well, and the optical density was measured using a luminometer (Turner T20/20). The experiment was repeated three times.

### 2.6. Serology Tests

HBsAg and HBeAg were detected using the double-antibody sandwich method with a COBAS e601 electrochemiluminescence spectrometer (Roche) at a lower limit of 1.1 COI (cut off index) for HBsAg and 1 COI for HBeAg. HBV DNA was examined by real-time fluorescence quantitative PCR using a Roche LightCycler 480 (Roche). The liver function indicators ALT and AST were measured using an enzymatic method with an ADVIA 2400 automated biochemistry analyzer (Siemens).

### 2.7. SPINK1 Test

Using human serum samples stored at -80°C, SPINK1 was detected with an SPINK1 enzyme-linked immunosorbent assay (ELISA) kit (Abcam) according to the manufacturer's instructions.

### 2.8. Statistical Analyses

Data were analyzed using SPSS 20.0, and the results are expressed as the mean ± standard deviation ($\overset{-}{x}\;\;$± s). Data comparisons between two groups were conducted using* t*-tests. Differences were considered significant at* P* < 0.05.

## 3. Results

### 3.1. Clinical Information for the Participants

The 248 patients infected with HBV were divided into three groups: 104 patients with CHB, 82 patients with LC, and 62 patients with HCC; 167 HBV-infected patients were tested positive for HBeAg and 81 negative for HBeAg.  Table 1 shows the clinical parameters and biochemical test results for the patients infected with HBV.

### 3.2. Increased Serum SPINK1 Levels in Patients Infected with HBV

**Figure 1:**
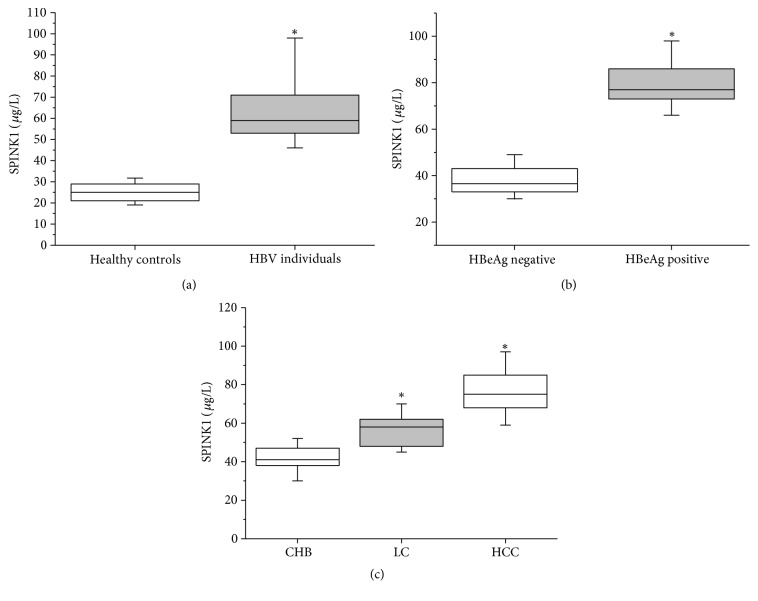
Serum SPINK1 levels in patients infected with HBV and healthy control participants detected by ELISA. (a) Comparison of serum SPINK1 levels between patients infected with HBV and healthy controls by ELISA. (b) Comparison of serum SPINK1 levels between patients testing positive and those testing negative for HBeAg. (c) Comparison of serum SPINK1 levels among patients with CHB, LC, or HCC. Box plots show medians with 25 and 75% and whiskers with 5 and 95% percentiles. *∗* indicates* P*<0.05.

The serum levels of SPINK1 were examined in the HBV group and the healthy control group using ELISA. The results showed a serum SPINK1 level of 63.3 ± 15.5 *μ*g/L for the healthy control group and 25.4 ± 6.7 *μ*g/L for the HBV group, a difference that was significant (P < 0.05; see Figure 1(a)).

Furthermore, the relationship between the serum SPINK1 level and HBeAg was analyzed. The results showed that the SPINK1 level in HBV-infected patients who tested positive for HBeAg (79.4 ± 9.4 *μ*g/L) was significantly higher than that of HBV-infected patients who tested negative for HBeAg (37.9 ± 5.9 *μ*g/L, P < 0.05, Figure 1(b)). No correlations were found between serum SPINK1 level and HBV viral load, ALT, or AST (data not shown).

CHB infection gradually develops into cirrhosis and ultimately leads to liver cancer. Thus, serum SPINK1 levels were compared between the groups of patients, and, according to the results, serum SPINK1 levels increased with continuous progression of HBV-caused diseases. Serum SPINK1 levels were 41.6 ± 6.6 *μ*g/L, 57.3 ± 8.3 *μ*g/L, and 77.9 ± 13.8 *μ*g/L in the CHB, LC, and HCC groups, respectively, and these differences were significant (P < 0.05, Figure 1(c)).

### 3.3. HBV Upregulates SPINK1 Expression at the Cellular Level

**Figure 2:**
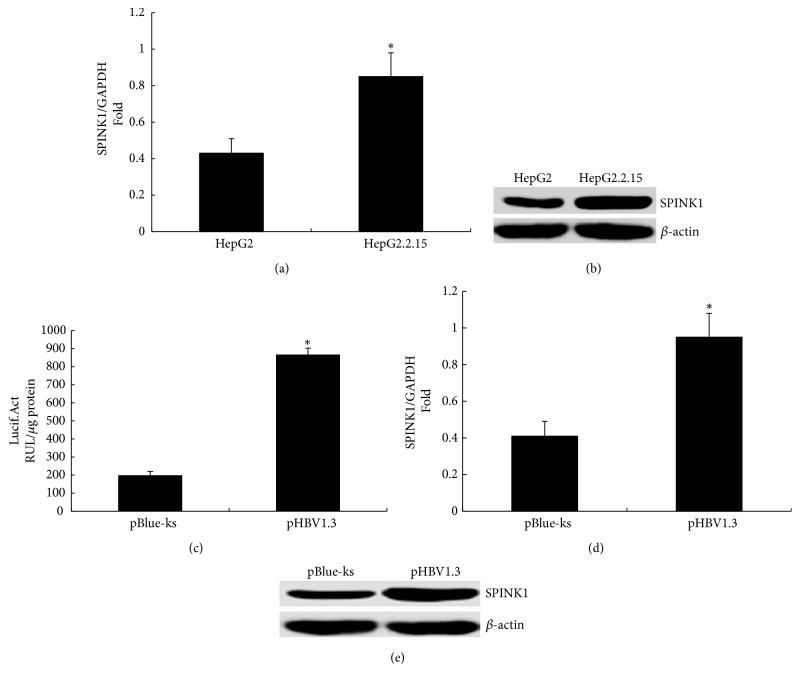
The effect of HBV on SPINK1 expression in cells. (a) The expression level of SPINK1 mRNA in HepG2.2.15 and HepG2 cells detected using RT-qPCR. (b) Expression of the SPINK1 protein in HepG2.2.15 and HepG2 cells was detected using western blotting. (c) Changes in luciferase activity at 48 hours after cotransfection of HepG2 cells with 0.2 *μ*g of SPINK1 promoter SPINK1-Luc and 0.6 *μ*g of HBV1.3 or its control plasmid pBlue-ks; the experiment was repeated three times. (d) SPINK1 mRNA expression was detected using RT-qPCR at 48 hours after transfection of HepG2 cells with 4 *μ*g of HBV1.3 or its control plasmid pBlue-ks. (e) SPINK1 protein expression detected using western blotting at 48 hours after transfection of HepG2 cells with 4 *μ*g of HBV1.3 or its control plasmid pBlue-ks. *∗* indicates* P*<0.05.

Expression of SPINK1 was examined in HepG2.2.15 cells with whole-HBV genome integration [14] and in control HepG2 cells using RT-qPCR and western blotting. The mRNA (Figure 2(a)) and protein expression levels of SPINK1 in HepG2.2.15 cells were higher than those in HepG2 cells (SPINK1/*β*-actin ratio of 0.91 in HepG2.2.15 cells and SPINK1/*β*-actin ratio of 0.43 in HepG2 cells, Figure 2(b)).

In addition, HepG2 cells were cotransfected with the HBV infectious clone pHBV1.3 and the SPINK1 promoter pSPINK1-LUC; cells cotransfected with the empty vector pBlue-ks and pSPINK1-LUC were used as the negative control. The results of the assay demonstrated that the activity of the SPINK1 promoter increased after transfection with pHBV1.3 compared with that in cells transfected with pBlue-ks; luciferase activities were 196.8 ± 22.7 RUL/*μ*g protein and 864.3 ± 38.2 RUL/*μ*g protein, respectively (Figure 2(c)), indicating that HBV activates the SPINK1 promoter. Moreover, RT-qPCR and western blotting revealed that the expression levels of SPINK1 mRNA (Figure 2(d)) and protein were increased after transfection with pHBV1.3 (SPINK1/*β*-actin ratio of 1.05 with pHBV1.3 transfection and SPINK1/*β*-actin ratio of 0.34 with pBlue-ks transfection, Figure 2(e)).

### 3.4. HBV Upregulates SPINK1 Expression through Its X Gene

**Figure 3:**
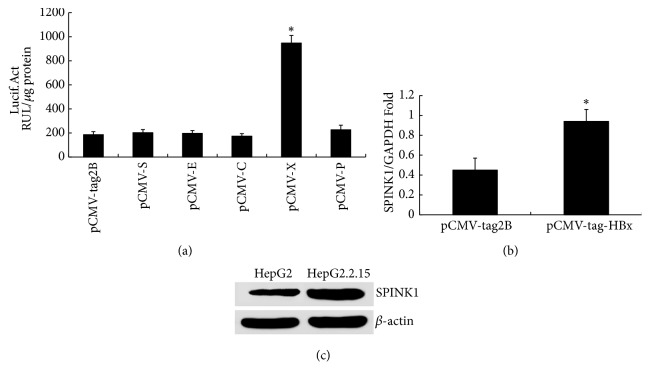
The effect of HBx on SPINK1 expression. (a) Changes in luciferase activity at 48 hours after cotransfection of HepG2 cells with 0.6 *μ*g of the eukaryotic expression plasmid containing each gene of the HBV genome (pCMV-S, pCMV-E, pCMV-C, pCMV-X, and pCMV-P) and 0.2 *μ*g of the SPINK1 gene promoter SPINK1-Luc or its control plasmid pCMV-tag2B; the experiment was repeated three times. (b) SPINK1 mRNA expression detected using RT-qPCR at 48 hours after transfection of HepG2 cells with 4 *μ*g of pCMV-X or its control plasmid pCMV-tag2B. (c) SPINK1 protein expression detected using western blotting at 48 hours after transfection of HepG2 cells with 4 *μ*g of pCMV-X or its control plasmid pCMV-tag2B. *∗* indicates* P*<0.05.

HepG2 cells were cotransfected with all of the individual plasmid eukaryotic expression vectors containing the HBV genome and the plasmid pSPINK1-LUC containing the SPINK1 gene promoter; cells transfected with the empty vector pCMV-2B were used as the control. HBx significantly upregulated the promoter activity of pSPINK1-LUC (Figure 3(a)).

A eukaryotic expression plasmid for the X gene, pCMV-X, was transfected into HepG2 cells, and cells transfected with the empty vector pCMV-tag2B were used as the control. Expression levels of SPINK1 mRNA and protein were significantly upregulated after transfection with pCMV-X, indicating that the HBV X protein enhances expression of SPINK1 mRNA (Figure 3(b)) and protein (SPINK1/*β*-actin ratio of 1.02 with pCMV-X transfection and SPINK1/*β*-actin ratio of 0.31 with pCMV-tag2B transfection, Figure 3(c)).

## 4. Discussion

SPINK1 is overexpressed in various solid tumors, such as gastric, breast, and colon cancers [15–18]. Marshall et al. screened for differentially expressed genes in normal liver tissue and HCC tissue using gene chips and found elevated SPINK1 expression in the latter [19]. Additionally, Li et al. examined SPINK1 expression in diseased tissues from patients with LC and those with HCC using immunohistochemistry and western blotting and found that expression of SPINK1 in patients with HCC was higher than that in patients with LC [6]. The current study investigated the serum SPINK1 levels of patients infected with HBV and healthy controls and found elevated SPINK1 serum levels in the former. Furthermore, the serum SPINK1 levels of patients who tested positive for HBeAg were significantly higher than those of patients who tested negative for HBeAg. We also compared serum SPINK1 among patients with CHB, LC, or HCC and found gradually increasing levels with the progression of HBV-related diseases. To elucidate the regulatory effect of HBV on SPINK1, we confirmed DNA chip results using RT-qPCR and western blotting and demonstrated that the levels of SPINK1 mRNA and protein expression were increased in HepG2.2.15 cells stably transfected with HBV.

HBV is a type of hepatotropic DNA virus that contains a partial double-stranded circular DNA molecule with four open-reading frames: S, C, P, and X [20]. To study the molecular mechanism of HBV with regard to regulation of SPINK1 expression, we employed the luciferase reporter gene system to detect the impact of pHBV1.3 and eukaryotic expression plasmids harboring its genes (i.e., pCMV-S, pCMV-E, pCMV-C, pCMV-X, and pCMV-P) on the activity of the SPINK1 promoter. We found that pHBV1.3 and its X gene upregulated the activity of the SPINK1 promoter and expression of SPINK1 at the mRNA and protein levels.

HBx is a regulatory factor encoded by the HBV X gene in HBV-infected cells which transactivates gene transcription and plays important roles in gene regulation, signal transduction, cell proliferation, and transformation, especially with regard to promoting the tumorigenesis of primary liver cancer, tumor cell invasion, and metastasis [21–24]. Studies have shown that SPINK1 participates in apoptosis by binding to epidermal growth factor receptor (EGFR), promoting tumorigenesis and progression [25]. Moreover, serum SPINK1 levels are closely related to tumor prognoses such as for ovarian, breast, bladder, and colon cancers, and SPINK1 overexpression serves as a marker of poor tumor prognosis [4, 5, 26, 27]. Therefore, HBV is likely to upregulate SPINK1 expression through its X gene to promote the development of liver cancer.

Thus far, serum markers such as alpha-fetoprotein isoforms, des-gamma-carboxy prothrombin (DCP), golgi protein 73 (GP73), glypican-3 (GPC3), and small RNA (miRNA) have been used for the clinical diagnosis of HCC [28]. The present study found SPINK1 to be associated with the progression of HBV-related diseases, and SPINK1 might become a new serum marker for HCC diagnosis. Because of the small number of specimens, however, the sensitivity and specificity of SPINK1 with regard to HCC diagnosis await further study.

In summary, we demonstrate that HBV upregulates the synthesis and secretion of SPINK1. These findings lay the foundation for elucidating the pathogenic and tumorigenic mechanisms of HBV to identify serum markers for HCC.

## Figures and Tables

**Table 1:** Clinical parameters and biochemical test results for the subjects enrolled in the study.

Characteristic	Healthy controls (n=124)	CHB	LC	HCC
		(n=104)	(n=82)	(n=62)

Sex (male/female)	90/34	78/26	60/22	48/14
Age	42.2 ± 14.6	40.5 ± 12.7	47.7 ± 15.3	55.8 ± 17.5
BMI (kg/m^2^)	23.5±1.4	25.3±1.6	24.7±1.5	24.4± 1.5
ALT (IU/l)	<30	206.4±283.7	52.7±76.8∗	59.9±81.5∗
AST (IU/l)	<30	146.7±200.3	65.9±65.1∗	92.7±88.6∗

n: number of subjects, CHB: chronic hepatitis B, LC: liver cirrhosis, HCC: hepatocellular carcinoma, BMI: body mass index, ALT: alanine aminotransferase, AST: aspartate aminotransferase. ∗ indicates *P*<0.05 compared with healthy controls.

## Data Availability

The data used to support the findings of this study are included within the article.
